# The Use of Simulated Body Fluid (SBF) for Assessing Materials Bioactivity in the Context of Tissue Engineering: Review and Challenges

**DOI:** 10.3390/biomimetics5040057

**Published:** 2020-10-29

**Authors:** Francesco Baino, Seiji Yamaguchi

**Affiliations:** 1 Institute of Materials Physics and Engineering, Department of Applied Science and Technology, Politecnico di Torino, Corso Duca degli Abruzzi 24, 10129 Torino, Italy; 2 Department of Biomedical Sciences, College of Life and Health Sciences, Chubu University, Aichi 487-8501, Japan

**Keywords:** bioactivity, simulated body fluid (SBF), biomaterials, apatite, bioactive glass, titanium, tissue engineering

## Abstract

Some special implantable materials are defined as “bioactive” if they can bond to living bone, forming a tight and chemically-stable interface. This property, which is inherent to some glass compositions, or can be induced by applying appropriate surface treatments on otherwise bio-inert metals, can be evaluated in vitro by immersion studies in simulated body fluid (SBF), mimicking the composition of human plasma. As a result, apatite coating may form on the material surface, and the presence of this bone-like “biomimetic skin” is considered predictive of bone-bonding ability in vivo. This review article summarizes the story and evolution of in vitro bioactivity testing methods using SBF, highlighting the influence of testing parameters (e.g., formulation and circulation of the solution) and material-related parameters (e.g., composition, geometry, texture). Suggestions for future methodological refinements are also provided at the end of the paper.

## 1. The Story of Simulated Body Fluid (SBF) Development

The assessment of bioreactivity, including bone bonding of implant materials, has been generally performed by an in vivo animal test, which was possibly started by Levert in 1829 [1], who studied gold, silver, lead, and platinum specimens in dogs. Although various types of artificial materials, such as steel, Co–Cr alloy, alumina, and titanium, have been implanted into bone defects, they were isolated from surrounding bone tissue due to fibrous connective tissue at the interface between the bone and implant. This was a normal protective reaction of the living body against foreign materials.

The first man-made material that bonded to bone without any fibrous tissue was the Na_2_O–CaO–SiO_2_–P_2_O_5_ glass reported by Hench and his colleagues in 1972 [2]. This discovery stimulated many researchers to develop various kinds of “bioactive materials”, such as sintered hydroxyapatite, β-tricalcium phosphate, Ceravital-type glass-ceramic containing apatite, and glass-ceramic A–W containing apatite and wollastonite, all of which directly bond to living bone [3]. Although these ceramic materials were successfully used as artificial middle ear small bones (ossicles), periodontal fillers, bone substitutes, iliac crest repair, and even in artificial vertebrae, their mechanical strength and fracture toughness were insufficient to use under load-bearing conditions, such as occurring in the femoral bone. It was, thus, greatly desired to confer bone-bonding capability on metals that are durable under load-bearing conditions. However, there was no clear indication of how to develop such materials.

When the various interfaces between bone and the bioactive materials were investigated, apatite layers were always found [4,5,6]. Based on these results, Kokubo and his colleagues speculated that materials forming an apatite layer on their surfaces are able, in principle, to bond to bone. They also speculated that this apatite formation is reproducible in vitro, and invented an acellular simulated body fluid (SBF) in which the ion concentrations and pH were nearly equal to these factors in human blood plasma in 1991 [7]. They found that the in vivo apatite formation of glass-ceramic A–W, Bioglass^®^, glass-ceramic Ceravital^®^, and sintered hydroxyapatite were successfully reproduced in vitro when these materials were simply soaked in SBF at 36.5 °C. They proposed that the bone-bonding capability of a given material could be evaluated by examining the apatite-forming capability on its surface in SBF.

The invention of SBF accelerated the development of bioactive materials since it catalyzed systematic study of apatite formation in various types of materials of various composition. Otsuki et al. investigated CaO–SiO_2_–P_2_O_5_ system glasses and reported in 1992 that CaO–SiO_2_ glass of a certain composition is able to form apatite in SBF [8]. Li et al. reported that SiO_2_ gel prepared by a sol-gel method formed apatite, also, in 1992 [9]. These results indicate that both CaO and P_2_O_5_, which are bone components, are not essential for apatite formation. Later, in the period 1994–2001, metallic oxide gels of TiO_2_, ZrO_2_, Nb_2_O_5_, and Ta_2_O_5_ were also found to form apatite in SBF [10,11,12,13]. These results indicated that certain kinds of hydroxyl groups, such as Si–OH, Ti–OH, Zr–OH, Nb–OH, and Ta–OH, are effective for inducing apatite formation in the body environment. This provided an indication of the general means of how to confer bone-bonding capacity on metals.

**Figure 1:**
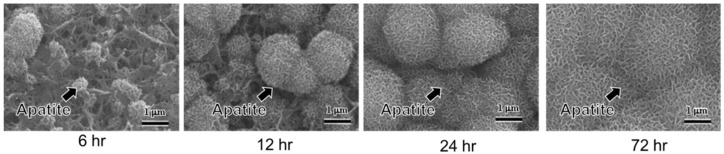
Apatite formation and growth on the surface of Ti subjected to NaOH and heat treatment by soaking in in simulated body fluid (SBF) (reproduced from [15]). The needle-like shape of bone-like apatite nanocrystals can be appreciated.

In 1996, Kim et al. reported that titanium and its alloys formed apatite on their surfaces in SBF when they had been simply immersed in 5 mol/L NaOH solution and subsequently subjected to a heat treatment at 600 °C, as shown in Figure 1 [14,15]. This apatite formation was attributed to a sodium titanate layer with abundant Ti–OH groups that formed on the metals. An in vivo animal test performed by Yan et al. showed that the NaOH-heat-treated titanium tightly bonded to bone via the apatite layer that had formed on the treated metal surface in the living body [16]. The NaOH and heat treatment was then applied to a porous Ti metal layer on the surface of an acetabular shell and femoral stem of a total artificial hip joint made of Ti–6Al–2Nb–Ta alloy, and this device has been in clinical use since 2007 [17]. A similar treatment was also applied to a spinal fusion device that has been in clinical use, without the need of any autograft, since 2018. This is in contrast with conventional Ti spinal fusion devices that require an autograft for fixation, since they cannot bond to living bone.

The SBF method, as the in vitro indicator of the bone-bonding capability of materials, came to be widely accepted by material scientists immediately after its invention. Since the original form of SBF lacked the SO_4_^2+^ ions that are present in human blood plasma, this was corrected in 1991. The corrected SBF is still richer in Cl^−^ ions and poorer in HCO^3−^ ions than human blood plasma. Thus, detailed studies to correct this difference were performed in 2003 and 2004 [18,19,20]. After careful assessment of its stability and the reproducibility of the apatite formation on synthetic materials, the corrected SBF with a refined recipe that allowed for easy preparation was proposed to the Technical Committee ISO/TC150 of the International Organization for Standardization (ISO). In 2007, the SBF was registered as ISO 23317, which is the standard solution for the in vitro evaluation of the apatite-forming ability of implant materials [21].

In 2006, Kokubo et al. summarized the correlation of in vivo bone bonding and in vitro apatite formation on various kinds of materials [22]. They described the quantitative correlation of apatite formation in SBF with in vivo bone bioactivity. On the other hand, they noted that there are a few exceptional cases in which this criterion is not valid, e.g., if the materials are highly resorbable or cytotoxic. β-tricalcium phosphate and natural calcite bond to living bone without forming an apatite layer on their surfaces. In contrast, natural abalone shell has apatite form on its surface in SBF, but does not bond to living bone. 

Some concerns on the validity of SBF were proposed by Bohner et al. and Pan et al. in 2009 and 2010 [23,24]. Bohner et al. pointed out “(i) the absence of proteins, whereas they are known to play an essential role in controlling apatite nucleation (nucleation inhibitors); (ii) the addition of *tris*(hydroxymethyl)aminomethane (Tris) to buffer SBF solutions; and (iii) the absence of any control for the carbonate content of the SBF solutions, although carbonates act as pH buffer in serum.” Pan et al. pointed out “bioactivity cannot be predicted from observed apatite forming ability simply on immersion in a so-called simulated body fluid” and suggested that the development of osteoconductive surfaces “can only be done through testing in vivo or, using osteoblasts under realistic conditions, in vitro”. Despite the limitations of SBF and these concerns, however, SBF has remained a powerful tool for predicting in vivo bone bonding ability. In 2014, Zadpoor reviewed 33 published studies available in the literature at the time, in all of which both the in vitro apatite forming ability and in vivo performance of two or more biomaterials were compared [25]. He concluded that in the clear majority of the studies (25/33 cases), the SBF immersion test proved successful in predicting the relative in vivo bioactivity of the materials. However, he suggested that “the details of the test protocols and the mechanisms of bioactivity of tested biomaterials should be carefully considered in the design of SBF immersion tests and in interpretation of their results”.

## 2. The Role of SBF Composition

As summarized By Nommeots-Nomm et al. [26] in a valuable review paper, a number of solutions have been proposed and used over the past 30 years to test the apatite-forming ability of biomaterials, including SBF, Tris buffer solution, dilute phosphate solutions and various cell culture medium formulations. Since 2007, the standard solution that is internationally adopted for in vitro bioactivity testing is the SBF formulation included in ISO 23317.

Perhaps the most common and oldest concern about the validity of this acellular solution is that SBF only reproduces the composition of the inorganic part of human plasma but does not contain any relevant bio-organic compounds, such as proteins, which are instead present in real biological fluids. This aspect was thought to be responsible for the differences found between biological apatite in natural calcified tissues and bone-like apatite layer formed on the surface of biomaterials upon soaking in SBF.

A series of studies published in 2003–2007 focused on this issue and investigated the influence of glucose [27], bovine serum albumin (BSA) [28], and cow’s milk [29] on the precipitation of a bone-like apatite layer from SBF. A similar behavior of BSA- and milk-containing SBF in terms of apatite formation rate was noted, but the studies were then discontinued by that group of researchers. Perhaps this was due to the problems of storage and preservation of the protein-modified solutions: if acellular SBF can be safely stored in a refrigerator at 5–10 °C for 1 month, preservation of BSA- or mill-containing SBF is obviously more difficult and shorter. Furthermore, sterile conditions are needed for both storage and testing. More recently, Magyari et al. [30] reported that the growth of an apatite-like layer on sol-gel bioactive glasses was lower if BSA-containing SBF was used compared to conventional SBF because calcium bonded to albumin. The inhibitory effect of BSA on hydroxyapatite during immersion in SBF was also observed by Zhao et al. [31]. 

Using cell culture media, such as Dulbecco’s modified Eagle medium (DMEM), has also been proposed as an alternative to SBF in order to better mimic “biological” conditions during in vitro bioactivity tests. Rohanova et al. [32], however, demonstrated that DMEM is unsuitable for such tests as it promotes the precipitation of calcium carbonate (CaCO_3_) but does not allow apatite formation; actually, this is not surprising as DMEM contains a high concentration of HCO^3−^ ions (ten times higher compared to SBF), which favors the precipitation of CaCO_3_ on the surface of bioactive glasses that release high amounts of Ca^2+^ ions during the test [33]. Furthermore, the use of DMEM requires the additional need to maintain sterile conditions during the bioactivity test.

Formation of calcium carbonate instead of or concurrently with apatite has also been observed to occur on the surface of bioactive glasses when “old” SBF formulations are used [18,19]. Thus, false negative results (i.e., a material may be incorrectly considered non-bioactive) could be due to following non-standard experimental procedures. Some papers in the literature—few, luckily—still suffer from such a mistake, as discussed in a recent review paper [34]; on the contrary, using the standard SBF formulation is key to obtain reliable results that can be robustly compared with those reported in similar experiments by different research teams.

## 3. The Role of Materials Chemistry

The chemical nature of the biomaterial considered indeed plays a key role on its bioactive properties; therefore, its apatite-forming ability upon immersion in SBF is strongly influenced accordingly. Unless properly treated, metallic materials are typically unable to induce the formation of apatite deposits on their surface in vitro; this topic will be specifically discussed in Section 5. On the contrary, some special glass formulations exhibit inherent bioactive properties. Hench [35] first proposed a five-stage process, conceptually similar to the mechanism of soda-lime glass corrosion, to explain the nucleation, growth and crystallization of bone-like apatite phases on the surface of silicate glasses upon immersion in aqueous solution:

(1) Glass-fluid ion exchange, involving the exchange of monovalent (Na^+^) and bivalent cations (Ca^2+^) from the glass with protons (H^+^) from the solution. This will lead to the formation of Si–OH (silanol bonds) on the glass surface;
(2) Rising of pH towards alkalinity, attack of Si–O–Si bonds by hydroxyl ions and formation of soluble silica Si(OH)_4_;
(3) Condensation and polymerization of silanol groups, with formation of a silica-rich amorphous layer (silica gel). The gel can absorb ions from the solution and, thus, be a “reactor” for apatite formation;
(4) Diffusion of calcium (Ca^2+^) and phosphate (PO_4_^3−^) ions through the silica gel and from the solution, leading to the formation of an amorphous calcium-phosphate film on the top of the silica gel;
(5) Crystallization of the calcium-phosphate layer and formation of hydroxyapatite, which is morphologically and crystallographically similar to bone bio-apatites.

**Figure 2:**
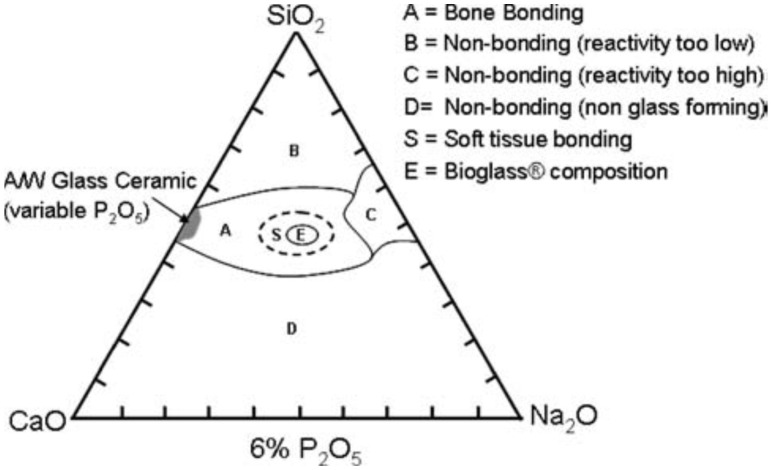
Compositional diagram used to design silicate bioactive glasses. Reproduced from [37] Hench, L.L. The story of bioglass. *J. Mater. Sci. Mater. Med.* **2006**, *17*, 967–978. Copyright 2006 Springer Nature.

Although the glass dissolution and apatite formation can last for a quite long period of time, the nucleation of the first crystals of hydroxyapatite is relatively fast and strongly depends on the molar percentage of SiO_2_ in the glass composition [36]. If the amount of SiO_2_ is below 53 mol.% in melt-derived glasses, hydroxyapatite crystallizes in less than 2 h and the glass can then chemically bond to living bone via this biomimetic interface; these glasses can also bond to soft collagenous tissues [37] (Figure 2). If the amount of SiO_2_ is increased up to 58 mol.%, the hydroxyapatite crystallization is slower and may take two days before happening. If the amount of SiO_2_ is higher than 60 mol.%, there was no evidence of hydroxyapatite crystallization after 1 month of immersion in SBF, although the formation of an amorphous calcium-phosphate layer was observed [36]. 

The ability to react and dissolve within SBF (or equivalent solutions) and induce apatite formation on the surface is strongly related to the glass network connectivity. The network of silicate glasses is formed by silica tetrahedra that are bonded together via Si–O–Si bonds [38]. Network modifiers such as Na_2_O, CaO, MgO, or K_2_O locally disrupt the glass network by breaking the Si–O–Si bonds and forming non-bridging oxygen [39]. In terms of glass dissolution kinetics, the less connected the glass network, the faster the glass dissolution [40]. This is the reason why melt-derived glasses with high silica content (above 60 mol.% [36]) are non-bioactive. The number of bridging oxygens of a silica tetrahedron can be expressed using the notation Q^n^, where n is the number of bridging oxygens and may range from 0 to 4. It was reported that the network of 45S5 glass (45SiO_2_–24.5CaO–24.5Na_2_O–6P_2_O_5_ wt.%), which is highly bioactive, is mainly composed of chains and rings of Q^2^ (almost 70% of tetrahedra have just two bridging bonds), while the remaining part is Q^3^ [41]. Starting from the glass composition, one can predict the network connectivity (i.e., the average number of bridging oxygens per each atom of Si), N_c_, as:

(1) $$N_{c}=\frac{4\left[{\mathrm{SiO}}_{2}\right]-2\left[M_{2}^{I}O+M^{\mathrm{II}}O\right]+6\left[P_{2}O_{5}\right]}{\left[{\mathrm{SiO}}_{2}\right]}$$

where $M_{2}^{I}O$ and $M^{\mathrm{II}}O$ are the mono- and divalent modifier oxides, respectively, that are present in the glass. Hence, the bioactivity of the material can be estimated knowing that, in order to be bioactive, a glass should exhibit a value of N_c_ in the range of 1.8 to 2.6 [41]. 

Although the multistep bioactivity mechanism described by Hench was initially developed for melt-derived 45S5 glass, it can be extended to most of bioactive silicate glasses produced either by melting or sol-gel process [42]. Some types of glass-ceramics (e.g., apatite–wollastonite (A–W)) were also reported to exhibit a similar behavior, forming a surface apatite layer in SBF albeit the silica gel layer was not detected [43]. It was also noted that the presence of crystalline phases can delay the formation of a surface apatite layer, but does not totally suppress the bioactivity in vitro even if crystallinity reaches 100% [44]. 

Borate-based glasses are more reactive than silicate systems in contact with aqueous media, such as SBF, and convert faster to hydroxyapatite than traditional bioactive glasses based on SiO_2_ as a network former. In principle, the bioactivity mechanism of borate glasses is similar to that of silicate glasses, but involves the formation of a borate-rich gel layer instead of silica gel. Fast dissolution is thought to be related to the structural changes in the glass network with increasing B_2_O_3_ content, since [BO_3_] trihedra cannot fully form a three-dimensional network compared to silica. Therefore, the dissolution-precipitation reactions occur continuously until the borate glass is fully converted to hydroxyapatite [45].

Phosphate-based glasses exhibit a typical tendency to quickly dissolve in aqueous solutions [46], which often precludes the formation of a stable surface apatite layer unless the glass composition is carefully designed to slow down the glass dissolution kinetics [47]. By adding modifiers (e.g., TiO_2_, CuO, or Fe_2_O_3_), the dissolution rate of phosphate systems can be varied from a few hours to months [48]. The low stability of such glasses is due to the asymmetry of [PO_4_] tetrahedra and the easy tendency to hydration of P–O–P bond [46].

Dissolution tests using phosphate dilute solutions or the Tris buffer solution are often performed as an alternative to immersion studies in SBF for borate- and phosphate-based glasses. 

## 4. The Role of Materials Geometry and Texture

The “geometrical” characteristics of samples can play a significant role on apatite formation in SBF, especially if the considered materials are inherently surface-reactive, such as bioactive glasses. In general, it is well known that the higher the specific surface area, the faster the ion-exchange mechanisms behind bioactive glass reactivity [49]. 

There is convincing evidence demonstrating that texture plays a predominant role over glass compositions as regards bioactive properties. It was found that, in non-porous melt-derived glasses, the content of SiO_2_ should be less than 60 mol.% to allow apatite formation on the surface in vitro and bonding to bone in vivo [50]; however, apatite can still form on the surface of SBF-treated glasses containing up to 90 mol.% of SiO_2_ if the material is obtained via sol-gel process due to inherent nanoporosity and high specific surface area available for glass-fluid interfacial reactions (above 10 m^2^/g vs. 0.10–0.30 m^2^/g for melt-derived products) [51]. This effect is further emphasized when a structure-directing agent is incorporated in the sol-gel process as a template for the development of an ordered mesoporous structure (specific surface area of the final calcined material above 100 m^2^/g) [52]. For example, hydroxyapatite crystals could form on mesoporous glass membranes with 80SiO_2_–15CaO–5P_2_O_5_ (mol.%) composition upon soaking in SBF for just 8 h, while the corresponding melt-derived material would be invariably considered an almost bio-inert material [53]. Even pure mesoporous silica (MCM-41 and SBA-15) was shown as able to form a surface apatite layer upon immersion in SBF due to its exceptional textural properties [54,55], but this process still requires too long a time to figure out a reliable bone-bonding application.

Sol-gel silicate glass-ceramics were also reported to exhibit good apatite-forming capability in SBF after 48 h regardless of the presence of crystalline phases [56], which instead often lead to a dramatic decrease of bioactivity in melt-derived materials [44]. 

In general, the extent of surface area, associated to both pore walls and outer surface, plays a pivotal role in bioactivity assessment. Sepulveda et al. [57] investigated the dissolution kinetics of melt-derived 45S5 glass powders having three dimensional ranges, i.e., fine (5–20 μm), medium (9–300 μm), and coarse (90–710 μm) sizes, during immersion in SBF. Overall, the dissolution results were directly correlated with the glass particle size in spite of an overlap of the size ranges, with the highest dissolution occurring for the smallest particles (5–20 μm). Accordingly, the pH increment towards alkalinity and apatite formation rate were faster as the particle size decreased; this trend (i.e., the finer the size, the higher the area, the higher the reactivity) was consistent with calcium dissolution and reduction in phosphorous concentration in the solution (phosphate “sequestration”), which is a proof of the apatite formation on the material surface.

Similar conclusions were reported in another study, [58], comparing the bioactive behavior of 45S5 glass in two well-distinct particle size ranges, i.e., 45–90 μm and 90–710 μm. Dissolution experiments were carried out in SBF at a fixed mass-to-volume ratio of 1.5 mg/mL. While the ion release tests shown in this publication did not reveal any significant difference between the ion concentration of the two samples, FTIR and XRD results suggested that apatite formed faster on the glass with a smaller size, with typical phosphate bands at 560 and 600 cm^−1^ being more pronounced after 1-day immersion, compared to coarse glass. The faster apatite-forming rate was associated with the higher specific surface area of fine glass particles, allowing faster ion exchange, and reaching supersaturation of the solution faster than large glass particles.

The same research group also elucidated how much the geometry of samples can affect the test execution and play a role from a methodological viewpoint [58]. They compared the method described in ISO 23317, which uses a fixed sample surface area-to-solution volume ratio, with another method, developed by the Technical Committee 4 (TC04) of the International Commission on Glass (ICG), which uses a fixed mass-to-volume ratio. Using the ISO method obviously led to different mass-to-volume ratios for different samples, depending on the glass particle size and surface area; however, if compositions with ultra-high surface area are used (above 100 m^2^/g like in sol-gel and mesoporous glasses [59]), this approach prescribes using a so low mass of glass that the experiments cannot be performed [58]. It is worth noting that the ISO standard was initially developed for solid samples with well-defined external geometry and shape (small tiles or cylinders), and Kokubo and Takadama [22], whose work was the basis of this standard, also recommended to further increase the volume of SBF for porous samples having an “effective” surface area (due to pore walls) higher than the outer one—but they did not quantify the extent of such an increment. Furthermore, the low mass-to-volume ratio deriving from the ISO method in case of powders or scaffolds could significantly delay the formation of apatite on the surface of samples. In order to overcome all these imitations, Macon et al. [58] recommended the use of a fixed mass-to-volume ratio of 1.5 mg/mL when bioactive glasses are tested in SBF in the form of powders or granules. 

**Figure 3:**
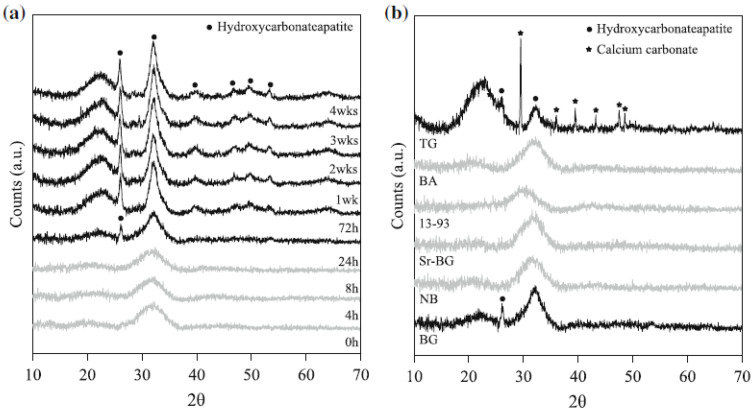
XRD spectra of (**a**) 45S5 glass particles (size 45–90 μm) after 0 to 4 weeks of immersion in SBF and (**b**) different silicate glass compositions after 24 h immersion in SBF. All of the glasses were produced by melting, except for the glass TG, which was obtained by sol-gel process. Reproduced from [58] Macon, A.L.B.; Kim, T.B.; Valliant, E.M.; Goetschius, K.; Brow, R.K.; Day, D.E., et al. A unified in vitro evaluation for apatite-forming ability of bioactive glasses and their variants. *J. Mater. Sci. Mater. Med.* **2015**, *26*, 115. Copyright 2015 Springer Nature.

These methodological aspects are crucial to avoid another problem that has been sometimes observed during immersion studies of bioactive glasses in SBF, i.e., the precipitation of CaCO_3_ instead of or concurrently with apatite on the surface of samples. This typically occurs if a too small volume of solution, or, in other words, a too high solid-to-liquid ratio, is used. As a result, the high concentration of Ca^2+^ ions in SBF may lead to CaCO_3_ precipitation [34]. From another perspective, it is apparent that the formation of CaCO_3_ is favored when glass particles with ultra-high surface area are tested if the volume of SBF is not properly selected. This means that the mass-to-volume ratio of 1.5 mg/mL suggested by Macon et al. [58] may still be too high to reveal the bioactive properties of ultra-porous or ultrafine materials, leading to an underestimation of the bioactivity or even false negative results due to CaCO_3_ precipitation. Early evidence of this problem was reported by Macon et al. [58] and Maclovic et al. [60], who observed the formation of CaCO_3_ on the surface of mesoporous and 45S5 nano-sized glass particles, respectively, after 1 day in SBF (Figure 3). Thus, a future optimization of experimental protocols for bioactivity assessment of highly-porous bioactive glasses should take into account this issue, rethinking the “optimal” volume of SBF to use with mesoporous and ultrafine glass micro-/nano-particles and probably shifting the mass-to-volume ratio below 1.5 mg/mL.

Furthermore, oxide substitutions in the glass composition may also carry the need for adjusting the mass-to-volume ratio used during immersion studies. When replacing an element with another of lower (e.g., Na with Li) [61] or higher atomic weight (e.g., Ca with Sr) [62] on a molar base, the molar weight of the glass changes. If a constant mass of glass is used for the experiments, the molar amount changes with composition, which has been shown to affect ion release during immersion studies [61].

The osseointegration of metallic implants also greatly depends upon the material structure and texture. Among the various parameters, surface roughness has received a great deal of attention in efforts to improve the bone-implant contact [63,64], and various techniques for turning, smoothing, blasting, and chemical etching, as well as electrochemical and deposition methods, have been developed to control the surface roughness of implants [65,66,67]. A highly roughened surface (Ra > 5 μm) is produced by plasma spraying and grid-blasting, while a moderately roughened surface (Ra = 1–5 μm) is produced by acid etching and anodization. A smooth surface (Ra < 1 μm) is obtained by a smoothing process performed by means of grit-paper [68].

Since the processing often alters not only the surface roughness, but also chemical factors, such as wettability and the surface charge caused by the residue of solid/solution and surface oxidation, a systematic study is needed to investigate the effect of roughness exclusively. Hacking et al. [69] compared bone apposition on four kinds of titanium that were polished, grit-blasted, plasma-sprayed with hydroxyapatite, or plasma-sprayed with hydroxyapatite, and masked with a very thin layer of titanium by means of physical vapor deposition (titanium mask). The titanium mask isolated the chemistry of the underlying hydroxyapatite layer without functionally changing its surface topography or morphologic features. When these were implanted into the femurs of dogs, the bone apposition was only 2.8% on as-polished Ti with an average roughness (Ra) of 0.09 μm, while it increased to 23% on grid-blasted Ti with an Ra of 3.64 μm. This was further increased to 73.6% on Ti with plasma-sprayed hydroxyapatite having an Ra of 5.58 μm. The bone apposition became 59.1% when the plasma-sprayed hydroxyapatite was masked with a thin Ti layer with an unchanged Ra. The enhanced osseointegration was understood to have occurred as a result of increased cell activity and mineralization capacity. The effect of the roughness on cell activity has been well summarized by many researchers elsewhere [63,64], and, therefore, this section focuses on the effect of the roughness on mineralization capacity, that is, the apatite formation on the materials that takes place in SBF.

SBF, as well as human blood plasma, is a solution supersaturated with apatite [7], and, thus, apatite formation on materials occurs as a result of the heterogeneous nucleation and growth of the apatite nuclei that spontaneously proceeds by taking up calcium and phosphate ions in SBF once the apatite nuclei have formed [7]. It should be noted that homogenous nucleation rarely occurs in SBF, but nevertheless has been reported in several papers [70]. The apatite nucleation that occurs is affected by both chemical factors such as hydroxyl groups, surface energy and surface charge, as well as physical factors, such as roughness. Chen et al. reported increased apatite formation on Ti, Zr, and TiZr alloys when the surface roughness of these metals was increased from Ra = 0.2 to 0.6 μm, followed by NaOH-heat treatment [71]. Sugino et al. reported that apatite formation was observed only on the internal surfaces of machined micro-grooves 500 μm deep and 500 μm wide on a Ti–15Zr–4Nb–Ta alloy subjected to heat treatment at 500 °C [72].

Since the utility of porous materials was discovered in the 1970s, numerous investigations on bone ingrowth into porous ceramics [73], polymers [74], and metals [75] have been performed. Among them, porous metals have been a particular focus due to their superior fracture and fatigue resistance properties that are required for load-bearing applications. The moderate porous structure obtained by sintering and additive manufacturing enables blood and cells to enter into the porous body, which results in bone ingrowth [76]. The bone ingrowth via the wall of a porous material, i.e., osteoconduction, is highly reliant on the pore size. The porous titanium fabricated by additive manufacturing with fully-interconnected pores in the range of 300–1100 μm exhibited a reasonable amount of bone ingrowth despite differences in porosity and the outer [77,78,79,80]. A remarkable increase in bone ingrowth was reported by Takemoto et al. in a study in which the porous titanium prepared by the sintering method, having the pore size of 300–500 μm, was subjected to NaOH, water, and heat treatment [81]. The treated porous body formed apatite throughout the inner pores in SBF and displayed significantly greater bone ingrowth depth and area after 8 weeks of implantation in a rabbit femoral condyle compared with an untreated porous body. They also showed that the treated porous titanium exhibited osteoinduction, that is, ectopic bone formation in muscle, when implanted into the dorsal muscle of a beagle dog [82]. This type of bone-forming porous titanium has been used in spinal fusion devices in Japan 2018. This device can be fixed to the surrounding bone without using any bone graft, which, in contrast, is required in the other devices currently in use.

## 5. The Role of Surface Treatments and Modifications in Materials That Are Initially Non-Bioactive 

There has been clinical demand in the orthopedic and dental fields for both biocompatibility and durable mechanical properties under load bearing conditions. The former is related to the surface characteristics of the material, while the latter is related to the bulk characteristic. The role of the surface modifications is to improve the surface characteristics of the materials so as to make them biocompatible with bone. Titanium metal (Ti) and its alloys intrinsically possess high biocompatibility and mechanical strength, and hence have been widely used as orthopedic devices and dental implants. This biocompatibility is attributed to the stable passive oxide layer that is a few nanometers in thickness that naturally forms on Ti substrate in air and even in water. However, this thin oxide layer does not always achieve stable fixation between the implant and bone for a sufficiently long period after implantation. In order to confer stable bone-bonding capability on Ti and its alloys, surface modification techniques related to hydroxyapatite, sand-blasting/acid-etching, anodic oxidation, chemical treatment and so on have been developed based on the strategies that improve the cell response and mineralization, and recently, the inflammatory response [83]. Among them, alkali-heat treatment and its derivative techniques such as calcification by chemical treatment have been developed in SBF, which is the focus of this section.

**Figure 4:**
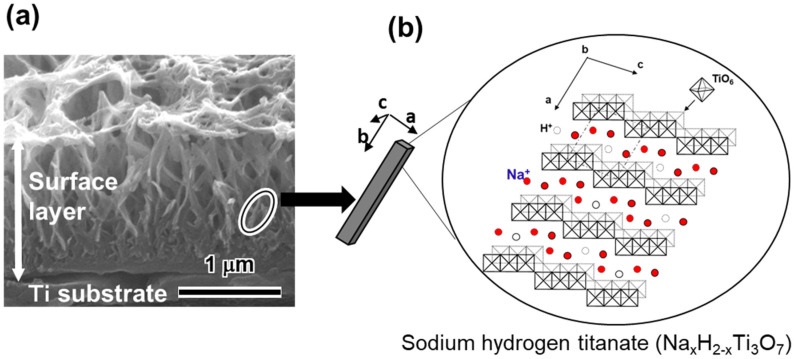
(**a**) Cross section SEM image of Ti subjected to NaOH treatment and (**b**) illustration of sodium hydrogen titanate structure. (Reproduced from (**a**) [15], (**b**) [85]).

A study showing apatite formation on titania gel in SBF [9] suggested that even Ti and its alloys formed surface apatite on their surfaces when abundant Ti–OH groups formed on their surfaces. Based on this suggestion, Ti was soaked in a 0.1–10 mol/L NaOH solution at 40–60 °C for 6–96 h [14,84,85] As a result, a lath-like layer developed on the metal surface with an increase in the concentration, temperature and soaking period. This layer was composed of nano-scale pillars of sodium hydrogen titanate (Na_x_H_2-x_Ti_y_O_2y+1_; 0 < *x* < 2 and *y* = 2, 3, or 4) that assume a layered structure as shown in Figure 4 [15,85]. It is noted that the structure of the resultant sodium hydrogen titanate can be changed by the conditions of the NaOH treatment. Guo et al. reported the formation of H_2_Ti_2_O_5_•H_2_O and Na_2_Ti_2_O_5_•H_2_O on Ti when the metal was subjected to hydrothermal treatment using NaOH solution at 0.15 MPa and 120 °C [86]. Possible compositions such as Na_2_Ti_9_O_19_, Na_2_Ti_6_O_13_, Na_2_Ti_2_O_4_(OH)_2_, Na_2_Ti_3_O_7_•nH_2_O, H_2_TiO_3_O_7_, H_2_Ti_2_O_5_, H_x_Ti_2−x/4_□_x/4_O_4_(□ = vacancy) have been proposed [87,88,89,90,91,92,93], while the mechanism of the precipitation of the sodium hydrogen titanate in NaOH solution is still under investigation. When the treated metals were soaked in SBF, they formed surface apatite when the metals were soaked in an NaOH solution higher than 0.5 mol/L. The apatite forming capacity of the treated metal increased with the increase of the concentration, temperature and soaking period in the NaOH treatment. The apatite formation was attributed to the abundant Ti-OH groups formed on the treated metals as a result of the exchange of the Na^+^ ions in the sodium hydrogen titanate for the H_3_O^+^ ions in SBF. This ion exchange proceeded rapidly, owing to the layered structure of sodium hydrogen titanate (Figure 4).

Although the simple NaOH treatment successfully does confer the apatite forming capability on Ti, the surface sodium hydrogen titanate layer that forms is mechanically weak, unstable, and the apatite formation capacity tends to be lost in the storage period before implantation or during the course of implantation [94]. Nishiguchi et al. reported the results of an in vivo test in which the detaching failure of NaOH-treated Ti was comparable to that of as-polished Ti, even after 16 weeks implantation in a rabbit tibia [94]. Thus, the treated Ti was subsequently heat treated at 600 °C for 1 h [95]. The heat treatment markedly increased the scratch resistance of the surface layer from 5 to 50 mN by transformation of the sodium hydrogen titanate into sodium titanate (Na_2_Ti_6_O_13_) and rutile without any apparent morphological change [15]. Interestingly, the sodium titanate also took on a layered structure and showed great capacity of ion exchange [95,96]. Thus, the treated metal again formed apatite on its surface in SBF. The apatite formation mechanism on the NaOH-heat treated Ti was revealed by X-ray photoelectron spectroscopy, transmission electron microscopy equipped with energy dispersive X-ray analysis and zeta potential measurement [97,98,99]: the exchange of Na^+^ ions and H_3_O^+^ ions produced not only Ti–OH groups on the metal, but also induced a local alkaline environment near the surface. As a result, the Ti–OH groups became negatively charged and combined with Ca^2+^ ions in SBF. As the Ca^2+^ ions accumulated, the surface became positively charged and combined with the HPO_4_^2−^ ions in SBF to form an amorphous calcium phosphate. This phase was metastable and eventually transformed into the stable, crystalline bone-like apatite.

It was not only the apatite formation that increased on the treated Ti, but also the cellular activity. Isaac et al. reported increased gene expression for bone protein markers such as alkaline phosphatase, osteocalcin, bone sialoprotein, and dentin matrix acidic phosphoprotein1, and also the expression of genes encoding the osteoblastic transcription factors Runx2, Dlx5, and Osterix [100]. It is worth mentioning that this gene expression upregulation was of the same degree on the treated Ti before and after apatite formation. 

Nishighchi et al. reported that when the Ti subjected to the NaOH and heat treatment was implanted into a rabbit tibia, it formed an apatite layer on its surface and tightly bonded to bone within 8 weeks [94]. They also reported an intramedullary rabbit femur model in which Ti rod implants subjected to NaOH and heat treatment displayed greater bone formation surrounding the implants and a significantly higher pull-out failure load than polished implants starting at 3 weeks [101]. The NaOH and heat treatment applied to the porous Ti layer of an artificial hip joint has been in clinical use in Japan since 2007. 

So et al. reviewed the long-term survivorship of the NaOH- and heat-treated total hip arthroplasty (THA) in 70 primary THAs, of whom 67 were available for follow-up in periods of 8–12 years [17]. Direct bone bonding, no radiographic signs of loosening, and a high survival rate of 98% at 10 years were reported. On the other hand, two joints had to be retrieved because of deep infection and periprosthetic femoral fracture, respectively [17]. In addition to bone bonding, the anti-bacterial and increasing bone density capacities are desirable, as these may prevent bone infection and fracture [102].

Such a multifunctional Ti may be realized by incorporating certain functional metal ions into the Ti surface by utilizing the layered structure of sodium hydrogen titanate. It is known that lithium (Li^+^), calcium (Ca^2+^), magnesium (Mg^2+^), strontium (Sr^2+^), zinc (Zn^2+^), and gallium (Ga^3+^) promote new bone formation, while silver (Ag^+^), copper (Cu^2+^), Zn^2+^ and Ga^3+^ exhibit antibacterial activity. Yamaguchi et al. and Kizuki et al. demonstrated that these ions can be incorporated into the Ti surface despite their valences when Ti is soaked in a solution containing these ions following the NaOH treatment owing to large ion exchange capacity of sodium hydrogen titanate [103,104,105,106,107]. The treated metal was subjected to heat treatment because the solution-treated metal again exhibited poor scratch resistance. The introduction of Ca^2+^ ions effectively confers apatite formation on certain kinds of Ti–Zr–Nb–Ta system alloys that are free cytotoxic elements. It is reported that the simple NaOH-heat treatment proved ineffective for these alloys [108]. When Ca^2+^ ions were incorporated into the Ti–36Nb–2Ta–3Zr–0.3O alloy surface by soaking in 100 mol/L CaCl_2_ solution after 1 mol/L NaOH treatment, followed by the heat treatment at 700 °C, calcium titanate was formed on the alloy. The treated alloy did not form apatite in SBF since the thus formed calcium titanate was too stable to release enough Ca^2+^ ions to induce apatite formation. A high capacity for apatite formation was conferred on the alloy by an additional hot water treatment at 80 °C for 24 h, which partially replaced the Ca^2+^ ions in the calcium titanate with H_3_O^+^ so as to form Ca-deficient calcium titanate [108]. When the treated alloy was implanted into a rabbit tibia, strong or poor bone bonding was observed on the alloy with or without the final hot water treatment, respectively [109]. Since the hot water treatment did not apparently change the crystal structure or surface topology, the strong bone bonding that occurred after the water treatment was attributed to apatite formation. Sr^2+^ and Mg^2+^ ions can be introduced into the calcium titanate without decreasing apatite formation when these ions are mixed in the second and final solution. The Ti with Sr- or Mg-containing Ca-deficient calcium titanate exhibited higher bone bonding capacity than the metal with Ca-deficient calcium titanate, especially at the early stages of implantation [110]. This increased bone bonding capacity was attributed to the increased cellar activity due to Sr^2+^ and Mg^2+^ ions that were released from the metal surface [103,105]. It was further demonstrated that Ag^+^ ions were controllably introduced into the Sr-containing calcium deficient calcium titanate by adding Ag^+^ ions in the final solution treatment [111]. The treated Ti with 0.2% Ag exhibited potent antibacterial activity (more than 99.9% reduction) against *E. coli* as well as apatite formation without any cytotoxicity. The bone bonding of the treated Ti was again confirmed by animal experiment.

These reports indicate that certain surface modifications developed by means of SBF successfully conferred bone bonding capacity on Ti, which in itself is a non-bioactive material. Furthermore, the surface treatments can be designed to provide additional key functions such as the promotion of new bone formation and antibacterial activity insofar as the apatite formation in SBF is maintained. This scheme for the development of multifunctional implants utilizing SBF should prove to be a promising way to produce desired implants in a timely manner.

## 6. The Role of Testing Mode: Static vs. Dynamic Conditions

If samples are available in a bulk form (i.e., tiles or discs), static testing in SBF is typically preferred [22]. If samples are tested in the form of fine powder or particulate, dynamic conditions have been proposed in order to better mimic the physiological conditions in the body, i.e., fluid recirculation [51]. In this regard, Macon et al. [56] proposed the testing of bioactive glass particles in SBF (fixed mass-to volume ratio of 1.5 mf/mL) under “standard” continuous agitation at 120 rpm in an orbital shaker during immersion at 37 °C. 

**Figure 5:**
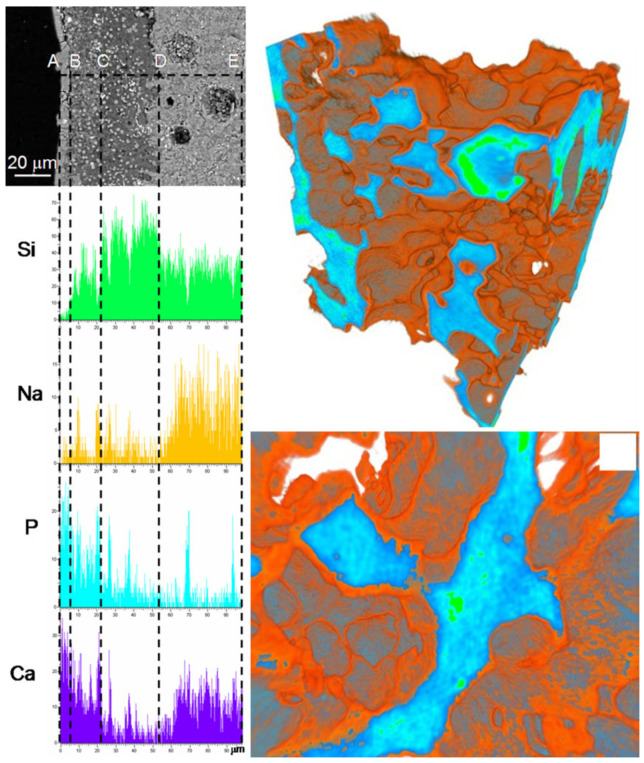
Results of in vitro bioactivity test on a silicate glass-ceramic scaffold: SEM-EDS analyses on a cross-section (**left**) and micro-tomographic three-dimensional reconstructions (**right**) after 14 days of immersion in SBF. Reproduced from [113] Miola, M.; Verné, E.; Vitale-Brovarone, C.; Baino, F. Antibacterial bioglass-derived scaffolds: innovative synthesis approach and characterization. *Int. J. Appl. Glass Sci.* **2016**, *7*, 238–247. Copyright 2016 John Wiley and Sons.

Zhang et al. [112] investigated the differences in terms of apatite-forming ability of 45S5 glass particles (size within 500–800 μm) during immersion in SBF for 48 h under both static (0.95 g glass in 40 mL of SBF) and dynamic conditions, with SBF circulation (0.95 g glass, flow rate 33 mL/min). Apatite was shown to form on the surface of glass particles in both testing modes, but exhibited different morphologies. Under static conditions, reactions layers were generally thicker, uneven, and included a distinct calcium-phosphate layer on the top of the silica gel layer. This multilayer appearance of the surface reaction layers is not always visible so clearly; it was well detected also on the pore walls of glass-ceramic scaffolds based on a SiO_2_–P_2_O_5_–CaO–MgO–Na_2_O–K_2_O–Ag_2_O glass system, as displayed in Figure 5 [113]. On the contrary, reactions layers formed under dynamic conditions were thinner and more uniform; furthermore, calcium phosphate inclusions were only observed in a single surface layer being mixed with silica gel [112].

A recent study reported the testing of gellan gum/sol-gel glass composite scaffolds in a perfusion bioreactor under SBF circulation [114]. The best results in terms of apatite formation on the walls of interior pores were obtained under direct SBF perfusion, while apatite only partially formed on the scaffolds immersed under static mode, with more crystals on the outer surface. This study suggests that bioactive glass conversion to apatite could be governed by the mass transfer rate and justifies future investigations for the development of effective in-vivo-like testing modes using bioreactors.

## 7. Beyond Bone-Related Bioactivity

Biomaterials community currently recognizes the use of SBF in immersion studies as a standard approach to evaluate the bioactivity of materials [22]. Indeed, the results of such in vitro assay, in terms of apatite-forming capability, are reliable only if the testing parameters are properly selected depending on the nature of the biomaterial tested: for example, the volume of SBF should be fixed according to the shape and geometry of materials, e.g., large pieces, particles, porous materials, etc. [34,58] Induction period of apatite formation in SBF enough for in vivo bone bonding should be discussed appropriately. On these points, further refinements of existing methods would need to be implemented in the future. 

In the light of recent advances in biomaterials science and bioengineering, a very basic issue deserves to be considered, i.e., the need for rethinking the meaning of the term “bioactivity”. In the broadest sense, a material is defined “bioactive” with reference to its ability of performing a specific function required to generate the most appropriate beneficial cellular or tissue response in a specific situation [115]. Of course, bioactivity implies biocompatibility, i.e., the material should perform its bio-function without eliciting any undesirable local or systemic effect in the host/biological environment [115]. Initially, bioactive materials were those materials that could bond to bone forming a tight and chemically-stable interface; this definition is still valid today, but a lot of experimental studies have clearly demonstrated that some materials—and, in some cases, exactly the same material, such as 45S5 glass—can be suitable to regenerate both bone and a range of soft tissues, thereby showing promise for use in nerve, cardiac and skin tissue engineering [116,117,118]. Therefore, the particular cell or tissue response deriving from the “bioactive process” should be referred to a broad range of applications that are not restricted only to bone-bonding ability. As the word “bioactive” impressively expanded its meaning, it should be always contextualized depending on the specific application. Hence, two strictly-interlocked questions emerge. The first: “Is the testing in SBF the most suitable in vitro method to investigate the bioactivity of materials?” The answer is indeed “Yes”, if the material goal is to promote bone healing (in this case, bioactivity = bone bonding and regeneration). On the contrary, if the intended application is in contact with soft tissues, the test in SBF could be misleading or meaningless—in fact, the apatite-forming capability could even be an undesired effect leading to soft tissue calcification. For example, it is estimated that the healing process of skin wounds lasts up to 14 days after the injury [119]. As a result, the dissolution rate of bioactive materials (e.g., glasses) applied for skin regeneration should be designed to somehow match this time period (ideally, the material should dissolve faster than soft tissue calcification occurs). The second question is: “What is the most suitable bioactivity test?” At present, the answer is not obvious, and, probably, will require the definition of “ad-hoc” testing methodologies depending on the specific application considered. This will be food for thought for further studies and discussions in the near future.
